# Conditional acceptance of emotion-recognition-enabled smart care devices for home-based rehabilitation and elderly care: a dual-perspective survey of prospective users and family caregivers

**DOI:** 10.3389/fpubh.2026.1849389

**Published:** 2026-06-03

**Authors:** Xiqiong Yi, XiaCheng Song, Jing Jin, Lu Sun, Huafeng Qu, Huirong Huang, Junfeng Zhu

**Affiliations:** 1 Dongguan Polytechnic, Dongguan, China; 2 Guangdong University of Science and Technology, Dongguan, China; 3 Xi'an Fanyi University, Xi'An, China; 4 Yunnan College of Business Management, Kunming, China; 5 Guangdong Industry Polytechnic University, Guangzhou, China; 6 Zhaoqing University, Zhaoqing, China

**Keywords:** older adult care, emotion recognition, human-in-the-loop, smart care devices, technology acceptance

## Abstract

Emotion-recognition-enabled smart care devices are increasingly proposed for home-based rehabilitation and elderly care, yet evidence on prospective user and family caregiver acceptance remains limited. This study investigated public attitudes, concerns, interaction preferences, and design expectations regarding such devices through a cross-sectional online survey of 506 adults in China. Respondents answered from either a prospective individual-user perspective (*n* = 339) or a family caregiver perspective (*n* = 167). Descriptive statistics, Top-2 Box summaries, and visual comparisons were used to analyze acceptance, scenario priorities, sensing preferences, data-sharing attitudes, and perceived device roles. Overall, respondents showed favorable but conditional acceptance. Privacy transparency, reliability, and recognition accuracy emerged as key adoption conditions, while concerns about emotional data misuse and machine accuracy remained salient. Participants prioritized home-based rehabilitation and health management scenarios, preferred wearable physiological sensing over camera-based monitoring, and favored human-in-the-loop interaction over fully automated responses. Family caregivers showed descriptively stronger endorsement than prospective individual users on several selected items, although the overall pattern was broadly consistent across groups and should not be interpreted as evidence of statistically significant subgroup differences. The findings suggest that emotion-recognition-enabled smart care devices may be more acceptable when designed around four principles: safety first, human-in-the-loop control, low-intrusion sensing, and bounded empathy. Because the sample consisted mainly of online adult respondents rather than actual older adult users, the findings should be interpreted as exploratory evidence on prospective acceptance rather than as direct evidence of real-world adoption.

## 1 Introduction

Population aging has become one of the defining demographic transformations of the 21st-century, intensifying pressure on health care systems, long-term care services, and family-based support structures (1, 2). In this context, home-based rehabilitation, aging in place, and community-embedded care have moved to the center of both policy and research agendas (3, 4) Smart care technologies, including smart home systems, remote monitoring tools, assistive devices, and health wearables, are increasingly positioned as promising responses to rising care demand, particularly where professional resources are limited and continuous in-person support is difficult to sustain (5). However, recent research makes clear that technical feasibility does not automatically translate into social acceptance (6). Studies on AI-based health technologies and health information technologies for older adults consistently show that adoption is shaped not only by perceived usefulness, but also by trust, usability, design fit, and broader social conditions (5, 7–9). In other words, the key question is no longer simply whether intelligent care technologies can be developed, but under what conditions they are likely to be accepted in everyday care environments (6, 9).

Within this broader landscape, emotion-recognition-enabled smart care devices represent a particularly important and sensitive emerging direction (10, 11). Unlike conventional health monitoring systems that focus mainly on physiological or behavioral indicators, these devices aim to infer affective states such as pain, discomfort, anxiety, fatigue, or emotional distress and to adapt their responses accordingly (11, 12). Such capabilities may be especially relevant in home-based rehabilitation and elderly care, where users often spend substantial periods without immediate professional supervision and where emotional states may directly affect safety, adherence, and quality of care (12–14). At the same time, the incorporation of emotion recognition introduces a more complex layer of ethical and practical concern (10). Recent studies on AI-led health technologies and smart home monitoring have repeatedly highlighted issues of privacy, security, autonomy, surveillance, and the continuing need for meaningful human oversight (13–15). Moreover, affective computing in rehabilitation and care is advancing rapidly at the technical level, yet translation into real-world use remains constrained by persistent challenges involving personalization, reliability, and data governance (12, 15, 16). Emotion-aware smart care systems therefore sit at the intersection of strong functional promise and heightened sociotechnical sensitivity (10, 13).

Despite this growing body of work, several important gaps remain in the user-acceptance literature (9). First, much of the existing research has examined broad categories such as AI health technologies, smart homes, wearables, or intelligent care systems (5, 6, 9, 17), while dedicated empirical evidence on user responses to emotion-recognition-enabled smart care devices remains limited (9, 10). Second, prior studies have often focused on general attitudes or behavioral intention (9, 18, 19), with less attention to the more design-relevant questions of scenario priorities, decision authority, sensing modality, emotional data sharing, and the acceptable scope of emotional memory. Third, most acceptance studies address only one stakeholder group at a time, typically either older adults or caregivers, even though care technologies are often assessed, recommended, and adopted within relational decision contexts (6, 18, 20). Recent work on intelligent care systems and caregiver-facing technologies suggests that family caregivers should be understood as independent adoption stakeholders rather than as peripheral observers (19–21). Taken together, these gaps indicate the need for a more focused, multi-stakeholder, and design-oriented investigation of how people evaluate emotion-recognition functions in smart care devices intended for domestic rehabilitation and elderly care.

To address these gaps, the present study conducted a dual-perspective cross-sectional online survey among adults in China to examine prospective responses to emotion-recognition-enabled smart care devices for home-based rehabilitation and elderly care. By collecting data from both a prospective individual-user perspective and a family caregiver perspective, the study moves beyond single-user acceptance models and investigates not only overall acceptance and perceived concerns, but also scenario priorities, design considerations, interaction and control preferences, sensing preferences, emotional data boundaries, and ideal device roles. In doing so, the study makes three main contributions: it frames acceptance as conditional rather than unconditional, treats family caregivers as independent adoption stakeholders, and translates acceptance boundaries into design-relevant implications for safety, human-in-the-loop control, low-intrusion sensing, and bounded empathy.

Research Questions

*RQ1:* What are the overall levels of acceptance and perceived concerns regarding emotion-recognition-enabled smart care devices among Chinese adults?

*RQ2:* Which care scenarios and design considerations are prioritized by respondents when evaluating emotion-recognition-enabled smart care devices?

*RQ3:* What interaction, sensing, and control preferences do respondents express regarding the use of emotion-recognition-enabled smart care devices?

*RQ4:* To what extent do individual users and family caregivers differ in their evaluations, and what boundaries do respondents place on emotional memory, emotional data sharing, and the ideal role of such devices?

## 2 Methods

### 2.1 Study design

This study employed a cross-sectional online survey to investigate public acceptance, concerns, interaction preferences, and design expectations regarding emotion-recognition-enabled smart care devices for home-based rehabilitation and elderly care. The survey adopted a dual-perspective framework in which each respondent self-selected one of two response perspectives at the outset of the questionnaire: (a) an *individual user perspective*, in which respondents evaluated the technology as potential future users themselves, or (b) a *family caregiver perspective*, in which respondents evaluated the technology as someone who might adopt the device on behalf of an older or health-impaired family member. This design was intended to capture both first-person experiential expectations and third-person care-oriented considerations within a single instrument, thereby enabling within-sample descriptive comparison of attitudinal patterns across the two stakeholder roles most directly relevant to the domestic adoption of smart care technologies. No experimental manipulation or intervention was involved.

### 2.2 Participants and recruitment

Participants were recruited through the paid sample service of Wenjuanxing, a commercial online survey platform widely used in mainland China. The questionnaire was distributed in simplified Chinese during a single data-collection window from February 10 to February 13, 2026. The final analytic sample comprised 506 valid responses, including 339 respondents (67.0%) answering from an individual user perspective and 167 respondents (33.0%) answering from a family caregiver perspective. Based on the cleaned dataset, the median completion time was 224 s (approximately 3 min and 44 s), and the mean completion time was 316.7 s.

### 2.3 Eligibility criteria and branching logic

Eligible participants were Chinese-speaking adults aged 18 years or older with potential interest in or relevance to home-based smart care devices, either as prospective individual users or as family members responsible for the care of an older or health-impaired relative. No additional restrictions were imposed on occupation, clinical status, or prior caregiving or device-use experience beyond the platform-based recruitment of adult respondents in mainland China. Group assignment was determined by the first survey item (Q1), which asked respondents to select their primary response perspective. Two items employed conditional display (branching) logic: Q3 (health conditions of the elderly family member under care) and Q12 (preferred decision authority after emotion recognition) were displayed only to respondents who selected the family caregiver perspective, as these items were structurally relevant only to the caregiving role. All remaining items were presented to the full sample regardless of perspective.

### 2.4 Survey development

The questionnaire was developed by an interdisciplinary research team with backgrounds in industrial design, product design, sports rehabilitation, software engineering, computer vision, and electronic information technology. The development followed an iterative process involving (a) a literature review of existing frameworks related to technology acceptance, affective computing, and elderly care robotics, drawing on constructs from the Technology Acceptance Model and the Unified Theory of Acceptance and Use of Technology (7, 22); (b) drafting of candidate items through a mixed strategy that combined newly designed items tailored to smart care scenarios with items adapted from established technology-acceptance and privacy-related frameworks (23, 24); and (c) refinement through internal expert review involving three reviewers with backgrounds in industrial design, sports rehabilitation, and intelligent software design, who examined the items for content relevance, clarity, and contextual appropriateness. The instrument was self-developed rather than adapted from a single validated scale, reflecting the absence of any existing standardized measure that specifically addresses user acceptance of emotion-recognition-enabled smart care devices for home-based rehabilitation and elderly care (6, 9, 18).

### 2.5 Survey instrument

Before the substantive items, respondents were introduced to emotion-recognition-enabled smart care devices through a brief textual description of hypothetical rehabilitation and elderly care scenarios, without reference to any specific commercial product or brand. The final questionnaire comprised 24 numbered items organized into five thematic sections; one additional embedded attention-check row was included within Q7. Section I (Q1–Q5) collected respondent profile information: response perspective (Q1; individual user vs. family caregiver), self-reported personal or health status (Q2; five categories), health conditions of the elderly family member under care (Q3; multiple choice, family caregivers only), age group (Q4; four categories), and highest education level (Q5; three categories). Section II (Q6–Q7) assessed acceptance and concerns: Q6 comprised five attitudinal items (perceived humanization, data-leakage concern, perceived usefulness, companion-like perception, distrust in machine accuracy), and Q7 comprised five substantive items assessing willingness to try, willingness to recommend, price sensitivity, accuracy prioritization, and the effect of privacy transparency on willingness to use, plus one embedded attention-check item instructing respondents to select “Strongly agree.” All items used a 5-point Likert scale (1 = Strongly disagree to 5 = Strongly agree). The attention-check item was used solely for response-quality screening and was excluded from all substantive statistical analyses and visualizations. Section III (Q8–Q9) assessed perceived importance of four application scenarios (Q8) and five device design considerations (Q9) on 5-point importance scales (1 = Very unimportant to 5 = Very important). Section IV (Q10–Q13, Q16–Q17) addressed interaction and control preferences through single-choice items covering target emotion for device response, preferred response to pain or discomfort, decision authority allocation (Q12; family caregivers only), preferred sensing modality, interaction orientation (function-oriented vs. emotion-oriented vs. balanced), and interaction style (progressive vs. stable vs. no preference). Section V (Q14–Q15, Q18, Q23–Q24) addressed emotional memory boundaries, data-sharing willingness, device design style, acceptance of art-toy appearance (Q15; 5-point Likert), and ideal device roles through a combination of single-choice and multiple-choice items. Four additional numbers (Q19–Q22) were automatically assigned by the survey platform to routing or display elements and did not collect substantive participant responses; they are therefore not reported in the present study.

### 2.6 Pilot testing and ethical considerations

Prior to formal deployment, the questionnaire was pilot-tested with a convenience sample of 30 respondents recruited through personal networks to assess item clarity, verify the functioning of the branching logic, and identify wording problems. Minor wording refinements were made on the basis of pilot feedback, and pilot data were not included in the final analytic sample. Regarding ethical safeguards, the study involved an anonymous, low-risk online survey without intervention or biological sampling. An informed consent statement was presented on the first page of the questionnaire, describing the purpose of the study, the voluntary nature of participation, the anonymous treatment of responses, and the intended use of the data for academic research. Respondents indicated consent before proceeding to the questionnaire. Ethical approval information is provided in a separate document for blinded review.

### 2.7 Data quality control

Survey responses were screened prior to analysis using multiple quality-control procedures. These procedures included an embedded attention-check item, response-time screening for implausibly short submissions, and manual review of clearly abnormal or logically inconsistent response patterns. After data cleaning, 506 valid responses were retained for the final analysis.

### 2.8 Data analysis

**Figure 1:**
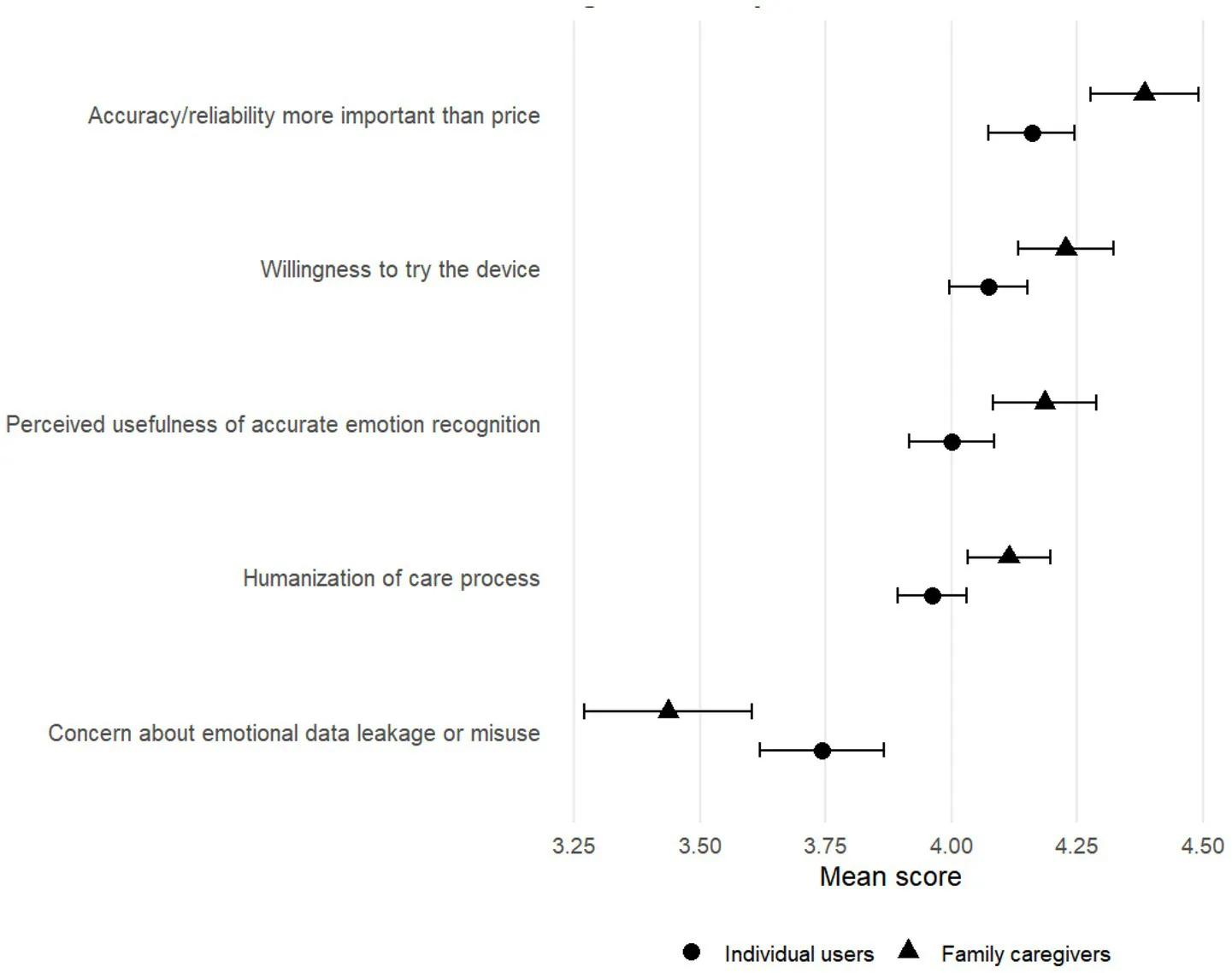
Comparison between individual users and family caregivers on selected key items. Points represent subgroup means, and horizontal error bars indicate 95% confidence intervals. The figure is intended to highlight directional differences between the two response perspectives.

**Figure 2:**
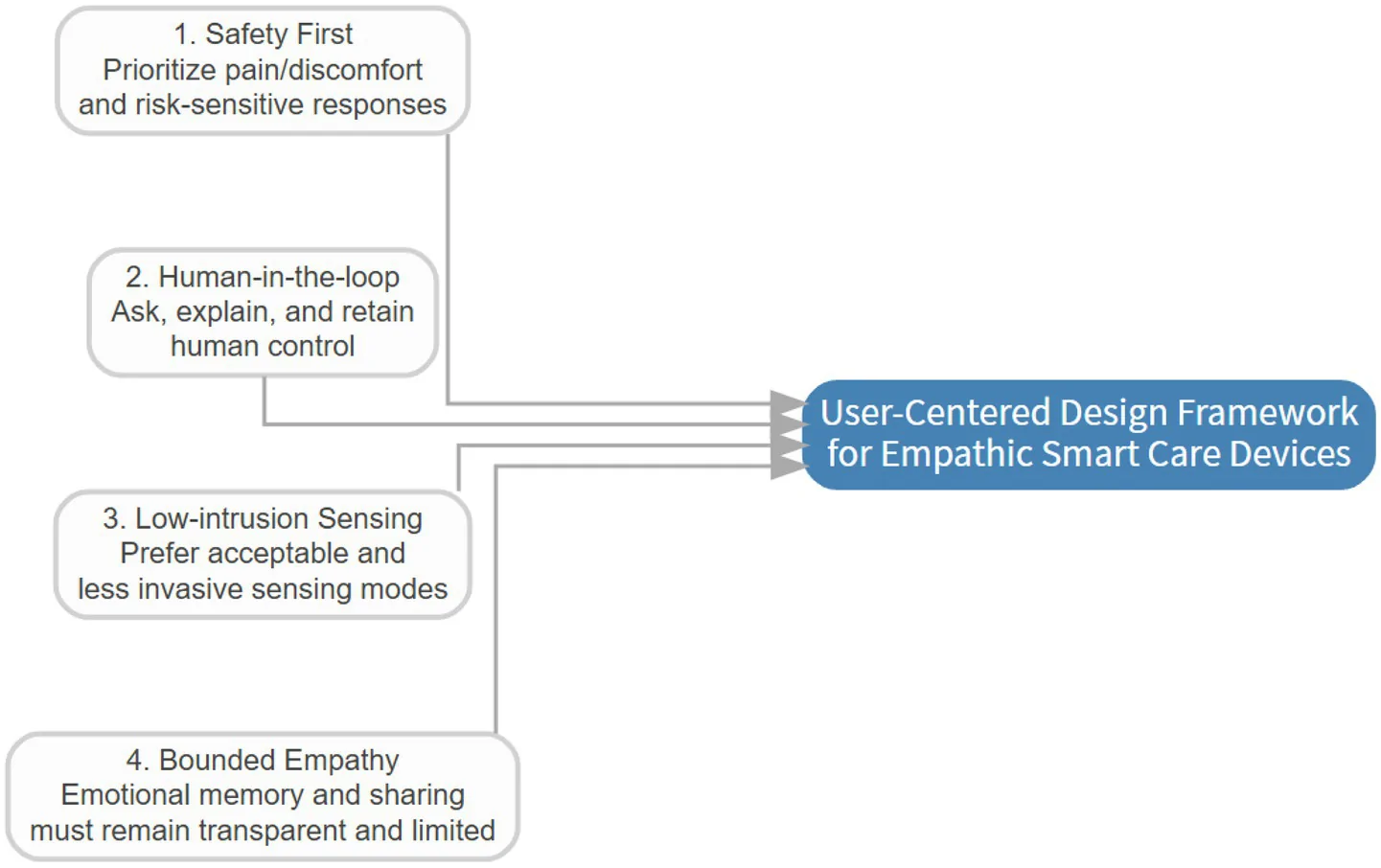
Design-implication framework for emotion-recognition-enabled smart care devices. This conceptual framework is derived from the empirical findings reported in Tables 2–4 and Figures 1, 3, 4. It synthesizes the major design implications of the study into four principles: safety first, human-in-the-loop control, low-intrusion sensing, and bounded empathy. This figure is not a statistical plot or validated model but an interpretive framework grounded in the descriptive survey results.

**Figure 3:**
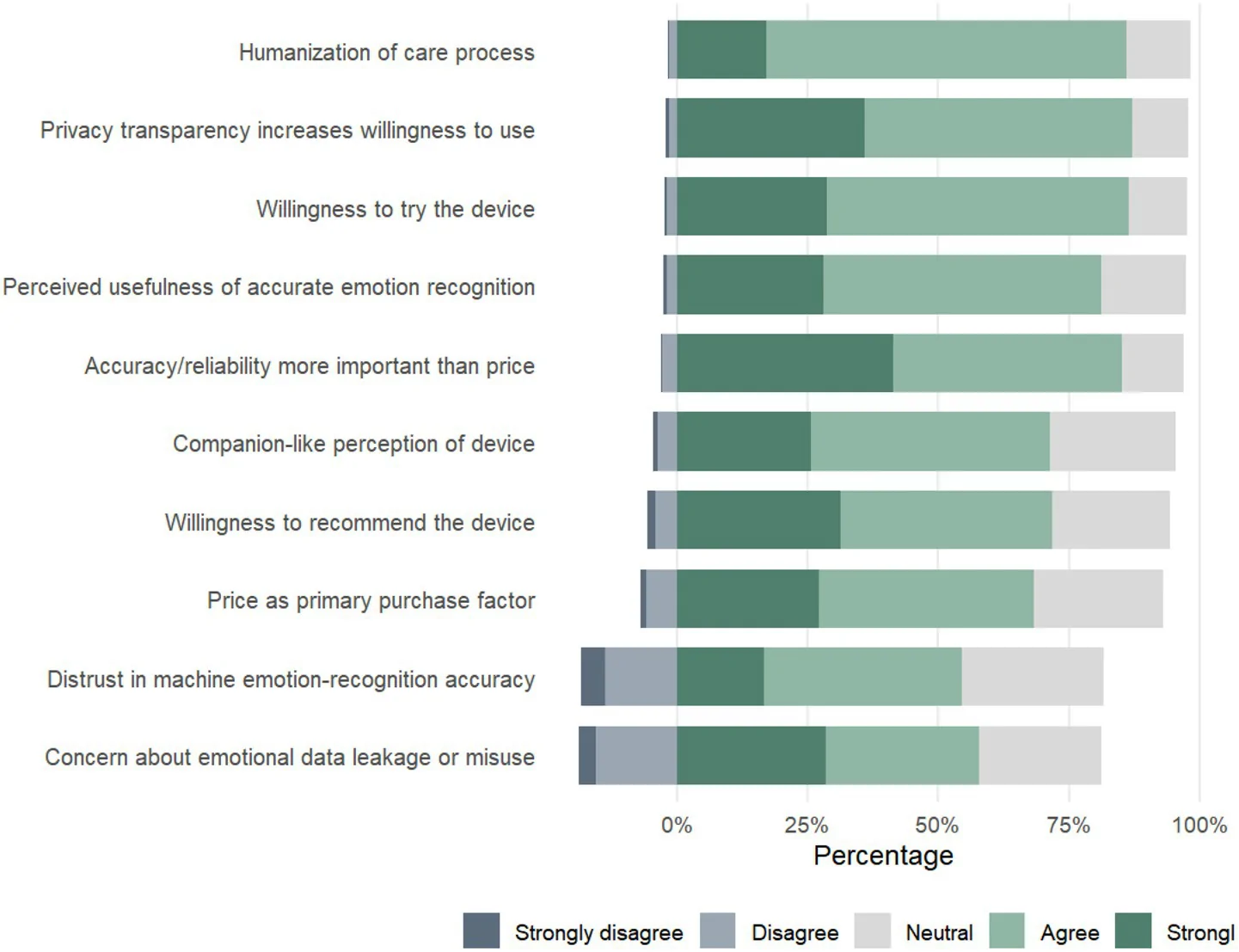
Diverging stacked bar chart of core acceptance and concern items. Percentages represent the distribution of responses across the five Likert categories for the core items in Q6 and Q7. The attention-check item was excluded. This figure visually summarizes the coexistence of positive expectations and perceived concerns regarding emotion-recognition functions in smart care devices.

**Figure 4:**
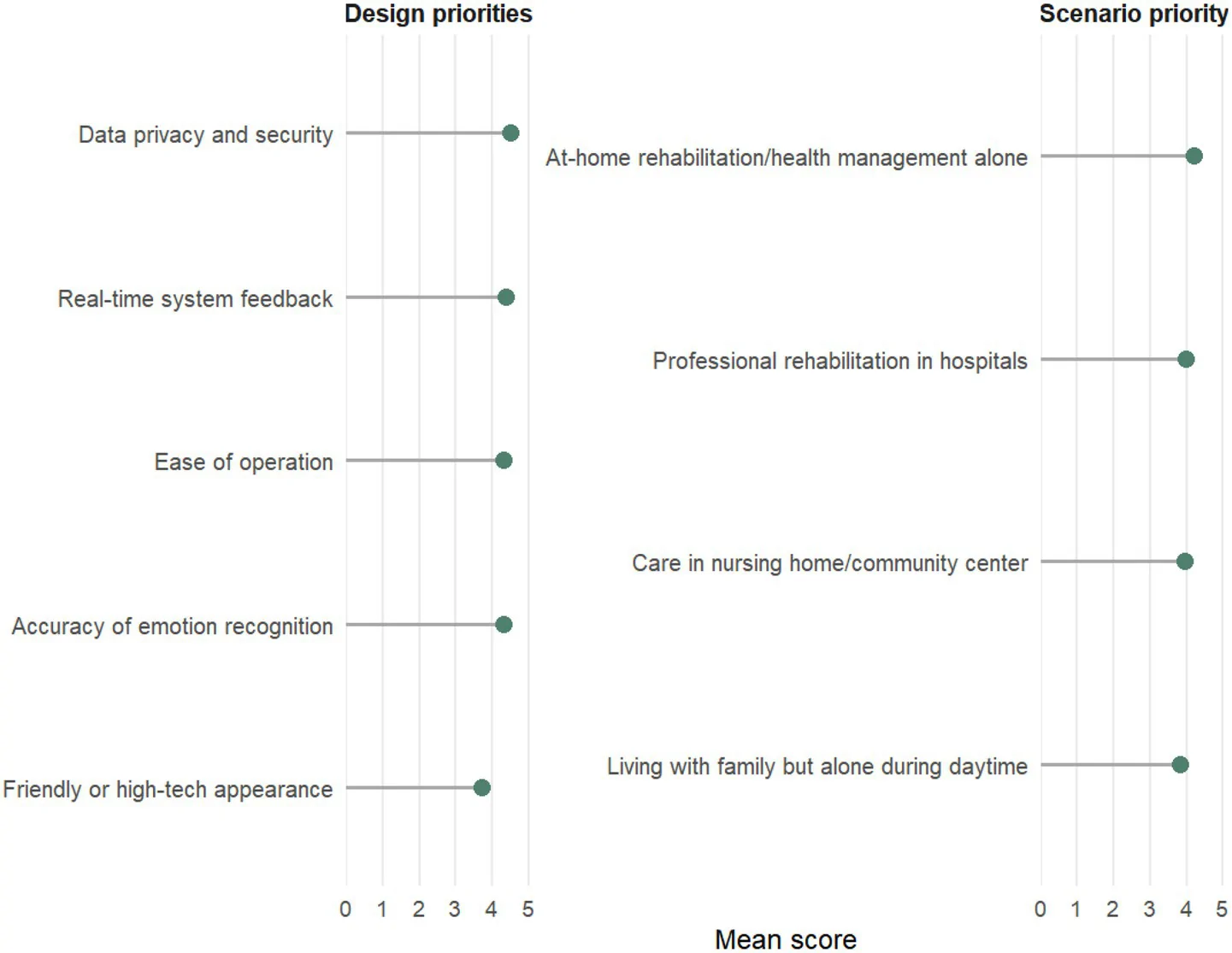
Priority ranking of scenarios and design considerations. Mean scores are based on five-point rating scales. The left panel summarizes the importance of key device-related considerations (Q9), whereas the right panel summarizes the perceived importance of different application scenarios (Q8). Higher values indicate greater perceived importance.

All analyses were conducted using R version 4.5.2 (25), with the *dplyr*, *tidyr*, *purrr*, *tibble*, *stringr*, and *forcats* packages for data processing, *ggplot2* for statistical graphics, and *DiagrammeR* for the conceptual framework diagram. The analytic strategy focused primarily on descriptive statistics, reflecting the exploratory and design-oriented aims of the study. For Likert-scale items (Q6, Q7, Q8, Q9, Q15), means, standard deviations, and Top-2 Box percentages were computed; Top-2 Box was defined as the proportion of respondents selecting a score of 4 or 5 (i.e., “Agree”/“Strongly agree,” “Important”/“Very important,” or “Somewhat like”/“Strongly like,” depending on the item), providing a readily interpretable index of positive endorsement. For single-choice categorical items, frequencies and percentages were reported. For multiple-choice items (Q3, Q18, Q24), each option was coded as a binary variable (1 = selected, 0 = not selected), and percentages were calculated as the number of respondents selecting each option divided by the total number who answered the item. Respondents were divided into two subgroups based on self-selected response perspective (Q1): prospective individual users (*n* = 339) and family caregivers (*n* = 167); between-group patterns on selected key items were examined descriptively through a comparison of subgroup means and 95% confidence intervals (Figure 1). Given the exploratory nature of the study, the unequal group sizes, and the design-oriented purpose of the subgroup comparison, formal inferential tests were not conducted; therefore, these subgroup patterns should not be interpreted as statistically significant differences. The four design principles presented in the conceptual framework (Figure 2) were derived inductively from convergent patterns across the descriptive results and are introduced here as an integrative summary of the survey findings; their broader implications are further addressed in the Discussion. Likert response distributions were additionally visualized using a diverging stacked bar chart (Figure 3), and mean priority ratings for scenarios and design considerations were displayed in a lollipop chart format (Figure 4).

## 3 Results

### 3.1 Sample characteristics by response perspective

A total of 506 participants completed the survey, of whom 339 (67.0%) responded from an individual user perspective and 167 (33.0%) from a family caregiver perspective (Table 1). Across the full sample, the majority were healthy young or middle-aged adults (75.3%), followed by young adults with care needs (15.0%), whereas patients in rehabilitation (4.0%), older adults aged 60 years and above (4.3%), and medical or rehabilitation professionals (1.4%) represented relatively small proportions. In terms of age, the sample was concentrated in the 18–30-year (42.9%) and 31–45-year (50.0%) groups. The two perspective groups showed some differences in demographic composition. Individual users were somewhat younger, with a higher proportion aged 18–30 (47.8%), whereas family caregivers were more concentrated in the 31–45-year range (57.5%). In terms of self-reported personal or health status, a larger proportion of respondents in the family caregiver group were classified under the category of older adults (10.2% vs. 1.5% in the individual user group). Educational attainment was consistently high across both groups, with 88.1% of the full sample holding an associate’s or bachelor’s degree.

**Table 1:** Sample characteristics by response perspective.

Variable	Category	Overall, *n* (%)	Individual user perspective, *n* (%)	Family caregiver perspective, *n* (%)
Response perspective	Individual user perspective	339 (67.0)	—	—
	Family caregiver perspective	167 (33.0)	—	—
Personal/health status	Healthy young/middle-aged adults	381 (75.3)	251 (74.0)	130 (77.8)
	Young adults with care needs	76 (15.0)	61 (18.0)	15 (9.0)
	Patients in rehabilitation	20 (4.0)	18 (5.3)	2 (1.2)
	Older adults (aged 60 and above)	22 (4.3)	5 (1.5)	17 (10.2)
	Medical/rehabilitation professionals	7 (1.4)	4 (1.2)	3 (1.8)
Age group	18–30 years	217 (42.9)	162 (47.8)	55 (32.9)
	31–45 years	253 (50.0)	157 (46.3)	96 (57.5)
	46–60 years	28 (5.5)	14 (4.1)	14 (8.4)
	61 years and above	8 (1.6)	6 (1.8)	2 (1.2)
Education	High school/technical secondary or below	28 (5.5)	20 (5.9)	8 (4.8)
	Associate’s degree/bachelor’s degree	446 (88.1)	300 (88.5)	146 (87.4)
	Master’s degree or above	32 (6.3)	19 (5.6)	13 (7.8)

Percentages for the two perspective groups were calculated within each subgroup (individual users: *n* = 339; family caregivers: *n* = 167). Percentages may not total 100.0 due to rounding.

### 3.2 Overall acceptance and perceived concerns

Descriptive statistics for overall acceptance and perceived concerns regarding emotion-recognition functions are presented in Table 2. Among the positively framed items, privacy transparency showed the highest endorsement (M = 4.20, SD = 0.74, Top-2 Box = 87.2%), followed by willingness to try the device (M = 4.12, SD = 0.71, Top-2 Box = 86.6%) and perceived humanization of the care process (M = 4.01, SD = 0.61, Top-2 Box = 86.0%). Strong support was also observed for prioritizing accuracy and reliability over price (M = 4.23, SD = 0.78, Top-2 Box = 85.2%) and for the perceived usefulness of accurate emotion recognition (*M* = 4.06, *SD* = 0.76, Top-2 Box = 81.2%). Intermediate levels of endorsement were observed for willingness to recommend the device (*M* = 3.96, *SD* = 0.92, Top-2 Box = 71.7%), companion-like perception of the device (*M* = 3.92, *SD* = 0.84, Top-2 Box = 71.3%), and price as a primary purchase factor (*M* = 3.88, *SD* = 0.91, Top-2 Box = 68.4%). At the same time, more cautious attitudes were evident for concern about emotional data leakage or misuse (M = 3.64, SD = 1.15, Top-2 Box = 57.9%) and distrust in machine emotion-recognition accuracy (M = 3.48, SD = 1.07, Top-2 Box = 54.5%). Overall, the findings indicate generally favorable acceptance of emotion-recognition functions, while also suggesting that such acceptance remained conditional on system reliability and transparent privacy protection.

**Table 2:** Overall acceptance and perceived concerns regarding emotion-recognition functions.

Section	Item	Mean	SD	Top-2 Box (%)
Q6	Humanization of care process	4.01	0.61	86.0
Q6	Concern about emotional data leakage or misuse	3.64	1.15	57.9
Q6	Perceived usefulness of accurate emotion recognition	4.06	0.76	81.2
Q6	Companion-like perception of device	3.92	0.84	71.3
Q6	Distrust in machine emotion-recognition accuracy	3.48	1.07	54.5
Q7	Willingness to try the device	4.12	0.71	86.6
Q7	Willingness to recommend the device	3.96	0.92	71.7
Q7	Price as primary purchase factor	3.88	0.91	68.4
Q7	Accuracy/reliability more important than price	4.23	0.78	85.2
Q7	Privacy transparency increases willingness to use	4.20	0.74	87.2

Top-2 box refers to the combined percentage of respondents selecting “Agree” or “Strongly agree.” The embedded attention-check item in Q7 was used only for response-quality screening and was excluded from the substantive analysis. Higher scores indicate stronger endorsement of the corresponding statement, except that the items on privacy concern and distrust of machine accuracy reflect reservations toward the technology.

Figure 3 illustrates the distributional structure of responses across the core acceptance and concern items. Items reflecting positive orientations, including willingness to try the device, privacy transparency increasing willingness to use, the perceived usefulness of accurate emotion recognition, and the view that accuracy or reliability is more important than price, showed clear rightward distributions, with responses concentrated in the “Agree” and “Strongly agree” categories. Humanization of the care process displayed a similarly strong positive skew, whereas companion-like perception of the device and willingness to recommend the device occupied a more intermediate position, remaining favorable overall but with a larger neutral share. In contrast, the two concern-related items, distrust in machine emotion-recognition accuracy and concern about emotional data leakage or misuse, showed visibly more dispersed profiles, extending further toward the disagreement side and containing larger neutral segments. Taken together, the figure suggests that acceptance of emotion-recognition functions was broadly positive, but not unconditional, as concerns about algorithmic reliability and emotional data privacy remained clearly present within the sample.

### 3.3 Scenario priorities and design considerations

Table 3 summarizes scenario priorities, design considerations, and interaction preferences across the full sample. With respect to application scenarios, at-home rehabilitation and health management was rated as the most important context (M = 4.20, SD = 0.66, Top-2 Box = 90.5%), followed by professional rehabilitation in hospitals (M = 3.98, SD = 0.84, Top-2 Box = 76.3%), receiving care in nursing homes or community centers (M = 3.95, SD = 0.88, Top-2 Box = 69.8%), and living with family but alone during the daytime (M = 3.83, SD = 0.91, Top-2 Box = 67.8%). Among device-related considerations, data privacy and security received the highest priority (M = 4.51, SD = 0.67, Top-2 Box = 92.3%), followed by real-time system feedback (M = 4.36, SD = 0.73, Top-2 Box = 89.1%), accuracy of emotion recognition (M = 4.30, SD = 0.67, Top-2 Box = 90.7%), and ease of operation (M = 4.30, SD = 0.70, Top-2 Box = 87.9%), whereas appearance was comparatively less emphasized (M = 3.71, SD = 0.90, Top-2 Box = 61.7%). In Panel B, pain or discomfort was the most frequently selected target emotion for device response (46.6%), and the most preferred response was a voice or pop-up inquiry that preserved human decision-making authority (50.2%), followed by immediate pause or safe reduction of intensity (40.1%). Among family caregivers, 66.5% indicated that the user should retain final decision authority after emotion recognition, compared with 21.0% endorsing automatic device handling. Wearable physiological sensors were the most preferred sensing modality overall (61.3%), and a progressive, increasingly personalized interaction style was favored by most respondents (73.1%).

**Table 3:** Scenario priorities, design considerations, and interaction preferences.

Panel A. Scenario priorities and design considerations				
Section	Item	Mean	SD	Top-2 box (%)
Q8	At-home rehabilitation/health management alone	4.20	0.66	90.5
Q8	Receiving care in a nursing home/community center	3.95	0.88	69.8
Q8	Living with family but alone during the daytime	3.83	0.91	67.8
Q8	Professional rehabilitation in hospitals	3.98	0.84	76.3
Q9	Accuracy of emotion recognition	4.30	0.67	90.7
Q9	Real-time system feedback	4.36	0.73	89.1
Q9	Data privacy and security	4.51	0.67	92.3
Q9	Ease of operation	4.30	0.70	87.9
Q9	Friendly or high-tech appearance	3.71	0.90	61.7

Panel B. Interaction Preferences		
Item	Response	*n* (%)
Target emotion for device response	Pain/discomfort	236 (46.6)
	Anxiety/irritability	162 (32.0)
	Fatigue/burnout	58 (11.5)
	Focus/engagement	24 (4.7)
	Pleasure/relaxation	26 (5.1)
Preferred response to pain/discomfort	Immediate pause or safe reduction of intensity	203 (40.1)
	Voice/popup inquiry, with the human making the decision	254 (50.2)
	Record and gently remind only, with manual control retained	49 (9.7)
Decision authority after emotion recognition (family caregiver subgroup only)	Device automatic handling	35 (21.0)
	The user	111 (66.5)
	Remote family members	21 (12.6)
Preferred sensing modality	Camera-based visual analysis	85 (16.8)
	Wearable physiological sensors	310 (61.3)
	Voice/prosody analysis	88 (17.4)
	No preference	23 (4.5)
Preferred interaction orientation	Utility-oriented: accurate recognition and rapid problem solving	281 (55.5)
	Emotion-oriented: emotional support and companionship	154 (30.4)
	Balanced	71 (14.0)
Preferred interaction style	Stable and consistent: predictable responses	117 (23.1)
	Progressive learning: increasingly personalized over time	370 (73.1)
	No particular preference	19 (3.8)

In Panel A, top-2 box refers to the percentage selecting the two highest response categories. In Panel B, percentages are based on valid responses to each item. The decision-authority item was asked only of respondents in the family caregiver subgroup (*n* = 167).

### 3.4 Visual comparison of scenario and design priorities

Figure 4 presents the mean priority ratings for device design considerations in the left panel and application scenarios in the right panel. On the design side, data privacy and security received the highest rating, followed closely by real-time system feedback, while accuracy of emotion recognition and ease of operation were also rated highly; by contrast, friendly or high-tech appearance was the lowest-ranked consideration. On the scenario side, at-home rehabilitation or health management alone emerged as the highest-priority application context, whereas professional rehabilitation in hospitals, care in nursing homes or community centers, and living with family but being alone during the daytime formed a somewhat lower cluster. The visual pattern across the two panels indicates that respondents prioritized functional reliability, privacy protection, and practical usability over appearance, while assigning the greatest value to contexts in which emotion-recognition functions could support more independent or care-intensive care situations.

### 3.5 Perspective differences in selected key judgments

Figure 1 compares mean ratings and 95% confidence intervals between individual users and family caregivers on five selected items. Across the four positively framed items, humanization of care process, perceived usefulness of accurate emotion recognition, willingness to try the device, and the view that accuracy or reliability is more important than price, family caregivers consistently showed slightly higher mean scores than individual users. By contrast, for concern about emotional data leakage or misuse, family caregivers reported a lower mean score than individual users, producing the most visible separation between the two groups in the opposite direction. The remaining items showed narrower gaps, although the overall pattern still favored somewhat stronger endorsement among family caregivers. Taken together, the figure suggests broad convergence between the two response perspectives, while also indicating that family caregivers were somewhat more supportive of the technology’s perceived benefits and somewhat less concerned about emotional data risks than respondents answering from the individual user perspective. These between-group patterns are presented descriptively; given the exploratory nature of the subgroup comparison and the unequal group sizes (*n* = 339 vs. *n* = 167), formal inferential tests were not conducted at this stage.

### 3.6 Boundaries of emotional memory and data sharing

Table 4 presents respondent preferences regarding device design, emotional data sharing, acceptance of emotional memory, and ideal device roles. In terms of physical design, a friendly companion-like style was the most preferred option (40.9%), followed by a minimalist high-tech style (32.4%) and a professional medical style (23.9%), whereas a fashionable trendy style received minimal endorsement (2.8%). Acceptance of an art-toy or artistic appearance was generally moderate to positive (M = 3.68, SD = 0.83), with 64.0% of respondents indicating that they somewhat or strongly liked this design direction. Regarding emotional data sharing, nearly half of respondents were willing to share emotional data with family members (49.0%), and 27.3% would share such data with doctors or caregivers, whereas 13.6% preferred to share only abnormal alerts and 10.1% were unwilling to share any emotional data. Most respondents also endorsed bounded forms of emotional memory, including remembering positive emotional moments for later encouragement (75.1%), remembering triggers of anger or sadness to avoid them later (73.9%), and remembering emotional cycles or patterns (64.4%), while only 4.7% preferred that nothing be remembered. Finally, the most frequently selected ideal device roles were invisible health manager (79.6%) and companion during solitude (77.5%), followed by bridge connecting family and clinicians (67.6%), strict rehabilitation coach (43.3%), and technological decoration in living space (28.1%), suggesting a clear preference for devices that combine practical support with relational sensitivity over those positioned as purely directive or decorative.

**Table 4:** Boundaries of emotional memory, data sharing, and ideal device roles.

Panel A. Design style, appearance, and emotional data sharing		
Item	Response	*n* (%)
Preferred device design style	Professional medical style	121 (23.9)
	Minimalist high-tech style	164 (32.4)
	Friendly companion-like style	207 (40.9)
	Fashionable trendy style	14 (2.8)
Acceptance of art-toy/artistic appearance	Strongly dislike	9 (1.8)
	Somewhat dislike	28 (5.5)
	Neutral	145 (28.7)
	Somewhat like	260 (51.4)
	Strongly like	64 (12.6)
Willingness to share emotional data	Willing to share with family members	248 (49.0)
	Willing to share with doctors/caregivers	138 (27.3)
	Willing to share only abnormal alerts	69 (13.6)
	Not willing to share any emotional data	51 (10.1)

Panel B. Acceptance of emotional memory and ideal device roles		
Item	Option	*n* (%)
Acceptance of emotional memory	Remember positive emotional moments and provide encouragement later	380 (75.1)
	Remember triggers of anger/sadness and avoid them later	374 (73.9)
	Remember emotional cycles or patterns	326 (64.4)
	Nothing should be remembered	24 (4.7)
Ideal role of smart care device	Invisible health manager	403 (79.6)
	Strict rehabilitation coach	219 (43.3)
	Companion during solitude	392 (77.5)
	Bridge connecting family and clinicians	342 (67.6)
	Technological decoration in living space	142 (28.1)
	Other	1 (0.2)

Percentages in Panel A are based on valid responses to each single-choice item. For Q15 (acceptance of art-toy/artistic appearance), the corresponding mean, standard deviation, and Top-2 Box percentage were *M* = 3.68, SD = 0.83, and 64.0%, respectively. Percentages in Panel B are selection rates for each option in the multiple-choice items; therefore, percentages within each item do not sum to 100.0.

Figure 2 synthesizes the empirical findings into a Design-Implication Framework for Emotion-Recognition-Enabled Smart Care Devices organized around four principles: safety first, human-in-the-loop, low-intrusion sensing, and bounded empathy. Safety first emphasizes prioritizing pain, discomfort, and other risk-sensitive responses; human-in-the-loop highlights the importance of asking, explaining, and retaining human control rather than relying on fully automatic intervention; low-intrusion sensing reflects the preference for acceptable and less invasive sensing modes; and bounded empathy underscores that emotional memory and emotional data sharing should remain transparent, limited, and clearly bounded. Rather than presenting a new statistical result, the figure serves as an integrative framework derived from the patterns observed across the preceding tables and figures. In this sense, it translates the survey findings into a concise design logic, highlighting safety, controllability, sensing burden, and bounded emotional responsiveness as recurrent design priorities emerging from the survey findings.

## 4 Discussion

### 4.1 Summary of principal findings

The present study shows that public responses to emotion-recognition-enabled smart care devices were broadly favorable, but clearly conditional rather than unconditional. Across the sample, respondents expressed substantial interest in trying such devices and viewed them as potentially useful and humanizing, yet these positive evaluations coexisted with persistent concerns about emotional data misuse and the accuracy of machine-based emotion recognition. At the same time, participants prioritized privacy protection, reliability, real-time responsiveness, and ease of use over appearance, preferred that the system remain supportive rather than fully autonomous, favored less intrusive sensing pathways, and accepted emotional memory only within clear boundaries. Taken together, these findings suggest that users are not simply endorsing “more intelligent” care technologies; rather, they are endorsing a bounded, trustworthy, and negotiable form of emotion-aware care in which assistance is welcomed only when it remains safe, transparent, and under meaningful human control.

### 4.2 Conditional acceptance rather than unqualified enthusiasm

A central implication of the present findings is that acceptance of emotion-recognition functions should be understood as conditional acceptance rather than generalized technological enthusiasm. This interpretation aligns closely with recent work on older adults’ perceptions of AI-based health technologies, which shows that prospective users often acknowledge potential benefits while simultaneously emphasizing privacy, usability, trustworthiness, and the irreplaceable role of human expertise and interaction (26). A recent systematic review likewise concluded that older adults’ acceptance of health information technologies is shaped not only by technology-related perceptions, but also by personal and social factors, underscoring that adoption cannot be reduced to usefulness alone (9, 27). In this context, our results extend the current literature by showing that, in the case of emotion-aware care devices, favorable attitudes do not displace concerns; instead, benefit expectations and perceived risks appear to coexist as parallel conditions of judgment.

### 4.3 Trust, transparency, and privacy governance as adoption conditions

The results further suggest that what drives acceptance is not simply the availability of affective intelligence, but whether that intelligence is embedded in a system that users perceive as trustworthy, transparent, and governable. Privacy transparency and the prioritization of accuracy and reliability both received particularly strong endorsement, while concerns about emotional data leakage and the limits of machine recognition remained salient. This pattern resonates with recent evidence from intelligent care system research showing that information trust is among the strongest predictors of perceived usefulness and behavioral intention, whereas stronger privacy-security demands may dampen acceptance if users believe the system is insufficiently governable (28). Our findings therefore push the discussion beyond a general claim that “privacy matters” by indicating that, in emotion-aware care contexts, transparent governance may be one of the few practical mechanisms capable of reconciling enthusiasm for intelligent support with concern about emotional data sensitivity.

### 4.4 Function-first adoption in high-need care contexts

Another important contribution of this study is the finding that respondents prioritized care-critical use contexts and core operational requirements over novelty, symbolism, or aesthetic appeal. At-home rehabilitation and health management, professional rehabilitation, and institutional care settings were all rated more highly than aesthetic considerations, while privacy security, real-time feedback, recognition accuracy, and ease of operation emerged as the dominant design priorities. This indicates that users primarily understand emotion-recognition-enabled smart care devices as functional care infrastructures rather than as lifestyle technologies or emotionally expressive consumer gadgets. That interpretation fits broader work on wearables and AI-enabled rehabilitation, which has increasingly emphasized that real-world adoption depends less on whether the technology is technically impressive than on whether it can be meaningfully integrated into actual monitoring, rehabilitation, and health management workflows (S. (29)). In this sense, our results challenge the assumption that emotional intelligence in care technologies will be accepted primarily because it feels more “human”; instead, they suggest that acceptance is grounded first in perceived care utility under conditions of vulnerability.

### 4.5 Human-in-the-loop as a user-demanded boundary

The preference structure observed in the interaction items indicates that human-in-the-loop control is not merely an abstract AI ethics principle, but a direct user demand in care-related emotion-recognition scenarios. Respondents most strongly wanted the device to respond to pain or discomfort, yet the most preferred response was not automatic intervention but a prompt that would ask the user and preserve human decision authority; likewise, family caregivers most often preferred that the user retain final control after emotion recognition. This pattern is highly consistent with current research on AI health technologies, which repeatedly shows that older adults and caregivers may value AI assistance while continuing to regard human judgment and interaction as indispensable (20, 26). In practical terms, our findings suggest that the legitimacy of emotion-aware smart care systems may depend less on whether they can act autonomously and more on whether they can detect, explain, and support without overruling the person being cared for.

### 4.6 Low-intrusion sensing and the preference for acceptable monitoring

The preference for wearable physiological sensing over camera-based visual analysis also carries important design implications. Rather than indicating a desire for maximal data capture, this pattern more plausibly reflects an effort by users to balance informational value against intrusiveness, visibility, and surveillance-related discomfort. Recent reviews of smart home monitoring and AI-based technologies for older adults have emphasized that privacy concerns are often inseparable from questions of autonomy, dignity, and the perceived purpose of continuous observation (10, 13). At the same time, contemporary work on wearables suggests that these systems are attractive precisely because they promise continuous support without always requiring conspicuous environmental surveillance (29). Our findings support this line of reasoning by suggesting that users are willing to accept sensing technologies when they are experienced as low-intrusion, physically manageable, and compatible with everyday care routines, rather than as ever-present forms of visual monitoring. This pattern is also consistent with (30), who found that acceptance of contactless monitoring technology in home-based dementia care varied by sensor type and care scenario among informal caregivers. Together, these findings suggest that acceptance of monitoring technologies in care settings depends not only on perceived usefulness, but also on sensor modality, intrusiveness, and the specific care purpose.

### 4.7 Family caregivers as independent adoption stakeholders

The comparison between individual users and family caregivers further suggests that care technology acceptance should not be modeled as a single-user phenomenon. Although the two groups showed broadly similar directional patterns, family caregivers tended to express somewhat stronger endorsement of the device’s potential benefits and somewhat lower concern regarding emotional data risks. This does not imply a fundamental divergence between the two groups, but it does indicate that smart care technologies are likely to be evaluated within a relational decision space in which the person being monitored and the person responsible for care may not weight values in exactly the same way. This point is especially relevant in light of recent work showing that guardians and family members constitute distinct acceptance subjects in intelligent eldercare systems (20, 31, 32). The contribution of the present study is therefore not only empirical but also methodological: by placing user and caregiver perspectives within the same survey design, it demonstrates the value of a multi-stakeholder acceptance lens for future care technology research.

### 4.8 From empirical patterns to design principles, limitations, and future directions

Finally, the four principles summarized in Figure 2, safety first, human-in-the-loop, low-intrusion sensing, and bounded empathy, provide a coherent design-oriented synthesis of the empirical results and help position this study within current debates on affective AI in care. Rather than treating emotional intelligence as a purely technical enhancement, these principles frame it as something that must be embedded within negotiated limits concerning risk, autonomy, sensing burden, and emotional data governance. At the same time, the study has several limitations: it was cross-sectional and based on self-reported attitudes toward hypothetical devices rather than long-term real-world use; it relied on an online panel sample that was relatively young and highly educated and therefore should not be interpreted as directly representing older adults themselves or people with lower digital skills; the online survey format may have underrepresented individuals with limited access to or familiarity with digital technologies; and the subgroup comparison remained descriptive rather than inferential, meaning that observed differences between prospective individual users and family caregivers should be interpreted as hypothesis-generating patterns rather than statistically confirmed group differences. Future research should therefore move toward prototype-based testing, longitudinal adoption studies, targeted studies involving older adults with direct care needs, and cross-cultural replications that examine whether the boundary conditions identified here remain stable across different sociotechnical and cultural contexts.

## 5 Conclusion

This study shows that public acceptance of emotion-recognition-enabled smart care devices is not unconditional, but shaped by a clear set of user-defined boundaries. Respondents generally recognized the potential value of such devices in home-based rehabilitation and elderly care, especially when they could improve safety, responsiveness, and care quality. At the same time, acceptance depended strongly on privacy transparency, recognition reliability, low-intrusion sensing, and the preservation of human decision authority. The comparison between individual users and family caregivers further suggests that these devices should be understood as multi-stakeholder technologies rather than single-user tools. Overall, the findings indicate that future smart care systems should not simply pursue more advanced emotion-recognition capabilities, but should instead be designed as trustworthy, bounded, and user-centered care technologies that support, rather than replace, human judgment and relational care.

## Data Availability

The raw data supporting the conclusions of this article will be made available by the authors, without undue reservation.
