# Correction: Conditional acceptance of emotion-recognition-enabled smart care devices for home-based rehabilitation and elderly care: a dual-perspective survey of prospective users and family caregivers

**DOI:** 10.3389/fpubh.2026.2023302

**Published:** 2026-10-26

**Authors:** Xiqiong Yi, XiaCheng Song, Jing Jin, Lu Sun, Huafeng Qu, Huirong Huang, Junfeng Zhu

**Affiliations:** 1 Dongguan Polytechnic, Dongguan, China; 2 Guangdong University of Science and Technology, Dongguan, China; 3 Xi'an Fanyi University, Xi'An, China; 4 Yunnan College of Business Management, Kunming, China; 5 Guangdong Industry Polytechnic University, Guangzhou, China; 6 Zhaoqing University, Zhaoqing, China

**Keywords:** older adult care, emotion recognition, human-in-the-loop, smart care devices, technology acceptance

The funding from the Specialized Innovation Project of Natural Science in Ordinary Colleges and Universities of Guangdong Province (Grant Number: 2025KTSCX376; project title: Research on Optimization of Human-Machine Interaction of Intelligent Rehabilitation Equipment Based on AI Machine Vision Emotion Recognition Technology) to Xiqiong Yi and the Dongguan Polytechnic University-level Fund Project (Grant Number: 2024a03; project title: Design of Motion Rehabilitation Assistive Devices Based on Intelligent Control Technology) to Xiqiong Yi was erroneously omitted.

The funding from the horizontal research project (Project No. GKY-2026 HX-052), associated with XiaCheng Song and Jing Jin, was erroneously included. Both references to this project in the Funding statement should be removed, including the project titles “Technical Service for the Development of Footwear Material Color and Product Visual Design Solutions Based on Footwear Material Technology and Fashion Trends” and “Technical Service for the Development of Shoe Material Color and Product Visual Solutions Based on Footwear Material Processes and Fashion Trends”.

The correct funding statement appears below.

“The author(s) declared that financial support was received for this work and/or its publication. This work was supported by the Specialized Innovation Project of Natural Science in Ordinary Colleges and Universities of Guangdong Province (Grant Number: 2025KTSCX376; project title: Research on Optimization of Human-Machine Interaction of Intelligent Rehabilitation Equipment Based on AI Machine Vision Emotion Recognition Technology) and the Dongguan Polytechnic University-level Fund Project (Grant Number: 2024a03; project title: Design of Motion Rehabilitation Assistive Devices Based on Intelligent Control Technology), both awarded to Xiqiong Yi. The funders had no role in the study design, data collection and analysis, decision to publish, or preparation of the manuscript.”

The original version of this article has been updated.

